# The Bidirectional Effect between Momentary Affective States and Exercise Duration on a Day Level

**DOI:** 10.3389/fpsyg.2016.01414

**Published:** 2016-09-21

**Authors:** Anna Schöndube, Martina Kanning, Reinhard Fuchs

**Affiliations:** ^1^Department of Sport Psychology, University of FreiburgFreiburg, Germany; ^2^Department of Sport and Exercise Science, University of KonstanzKonstanz, Germany

**Keywords:** ambulatory assessment, ecological momentary assessment, mood, physical activity, health behavior

## Abstract

Despite the well-documented positive effect of exercise on health outcomes, most people do not succeed in exercising regularly. In addition to several other influences, affective states seem to support exercise participation. Associations between exercise and affect have been shown in the laboratory. However, the dynamic relation between affect and exercise in daily life is not yet well-understood. The objective of this study was to investigate the bi-directional effect of momentary affective states on naturally occurring exercise and vice versa in healthy participants in real-life environments by applying an ecological momentary assessment design. We hypothesized that (1) exercise duration is positively associated with affective states on a day level, (2) affective states in the morning predict subsequent exercise duration, and (3) exercise duration predicts affective states in the evening on that respective day. Data from *N* = 60 students aged between 19 and 32 years were analyzed. Affect and exercise duration were assessed daily over a period of 20 days via an electronic diary. Multilevel analysis revealed that positive affective valence was positively associated with exercise duration (*p* = 0.003) on a day level. In addition, the more the participants exercised that respective day, the better and more content they felt in the evening (*p* = 0.009). Energetic arousal in the morning significantly predicted subsequent exercise duration (*p* = 0.045). The findings indicate that it would be worthwhile to focus more on within-subject analyses when analyzing the dynamic relation between affect and exercise. Furthermore, affective states should be taken into account in creating effective interventions to foster exercise behavior and enhance maintenance.

## Introduction

Most people do not reach the recommended volume of exercise, even though the health-enhancing effects are well-documented (Lee et al., 2012). Many factors have been examined as influencing factors on exercise behavior (Fuchs et al., 2012). Recent research has focused on state variables such as affect (Jekauc, 2015). An understanding of the dynamic relation between affective states and exercise may help to create effective exercise promotion programs. Ample evidence shows positive associations between positive affective states and exercise (Berger and Motl, 2000). However, most previous studies have investigated this association in the laboratory (Liao et al., 2015), whereas studies about the association between affective states and physical activity are rare, and findings are contradictory (Kanning et al., 2013; Liao et al., 2015). In addition, in attempting to understand the dynamic relation between these two variables, researchers have seldom focused on within-subject analyses, which have the advantage of yielding knowledge about dynamic processes rather than just about between-person differences. This study uses an ambulatory assessment approach to analyze the bi-directional effect between affective states and physical activity, especially naturally occurring exercise, in real life in healthy participants.

The effect of physical activity or exercise on affect has often been shown in the laboratory, which allows a controlled design (Ekkekakis and Petruzzello, 2000). However, these studies lack external validity. A few recent field studies have tried to answer the question of whether affective reactions are associated with physical activities in real life (e.g., Dunton et al., 2009, 2014; Schwerdtfeger et al., 2010; Kanning et al., 2013; von Haaren et al., 2013). The authors of these studies chose ecological momentary assessment (EMA) as a suitable method to collect real-time data in daily life (Shiffman et al., 2008). EMA involves having test subjects wear a tool in daily life that beeps and prompts them to complete an electronic diary on their phone.

In a review, Liao et al. (2015) systematically examined the short-term associations between affective states and subsequent acute free-living physical activity as well as the short-term associations between physical activity and subsequent affective states. Most of the EMA studies included in the review that examined the association between affect and subsequent physical activity found a positive association between positive affective valence and physical activity, whereas evidence of negative affective valence predicting physical activity was inconclusive. A study by Dunton et al. (2014) succeeded in additionally showing that energetic arousal positively predicts physical activity in children. Some studies examined in the review of Liao et al. (2015) also found an increase in energetic arousal and a decrease or an increase in calmness following physical activity. The mixed results of the association between physical activity and calmness might be explained by the length of the time interval between the assessment of both variables. When measuring calmess within 1 h after physical activity, a decrease in calmness has been shown (Kanning, 2013; Kanning et al., 2015). With longer time intervals an increase in calmness can be detected (Gauvin et al., 1996).

In conclusion, evidence suggests that positive affective valence and energetic arousal predict subsequent physical activity and that physical activity leads to positive affective valence and may affect calmness. However, most of the studies examined in the review by Liao et al. (2015) defined physical activity as activity in daily life, not solely activity executed for the purpose of exercising. Carels et al. (2007), for example, investigated the association between affect and exercise and found that affect in the morning is associated with an increased likelihood to exercise that day in obese dieters. They also found that positive affective valence following exercise is higher when the duration and intensity of the exercise are higher.

As demonstrated above, there is reason to assume that there is an association between affective states and exercise in real life. Understanding this association may be important for creating effective exercise promotion programs. Positive experiences with exercising might positively affect long-term maintenance (“outcome experiences”; Fuchs et al., 2012). A recent study by Jekauc (2015) has shown that inducing positive affect during exercise has an effect on long-term maintenance, indicating that positive affective valence plays an important role in exercise behavior prediction. Affecting attitudes through affective messages has been shown to lead to a stronger increase in exercise participation than affecting attitudes through health-related messages (Conner et al., 2011). In sum, positive affective valence can, if associated with exercising, be considered one of the strongest factors contributing to the maintenance of exercise behavior.

Empirical results indicate that there is an association between affect and physical activity in real life. We would like to broaden the evidence by testing whether there is also a bi-directional association between momentary affective states and freely occurring daily exercise participation in healthy participants. Unlike previous studies investigating daily life activities such as stairway climbing or vacuuming, our study aims to gain insights into planned sport activities (encompassing either classical “sports” or health-related physical exercise). We are interested in whether naturally occurring sport and exercise behavior is related to affective states. To avoid retrospective distortions and to observe real-life effects, we collected our data using electronic diaries (EMA). Our primary research hypothesis, developed on the basis of previous findings, was that (1) a participant would exercise more on days on which he or she reported higher mean positive affective valence, higher mean energetic arousal, and higher mean calmness. With regard to an additional focus on the temporal relationship between affect and naturally occurring exercise, we assumed in our second hypothesis that (2) high positive affective valence, high energetic arousal, and high calmness in the morning would predict longer exercise duration on that day. In our third hypothesis, we expected that (3) the more the participant exercised that day, the higher would be the positive affective valence, energetic arousal, and calmness reported by that participant in the evening.

## Materials and Methods

### Participants

Out of 75 participants, *N* = 60 participants provided sufficient data to be included in the analysis. We excluded 15 participants because they did not engage in any exercise during the 4 weeks of data collection or more than 50% of their values were missing or their data could not be analyzed due to technical problems. The sample included participants aged between 19 and 32 years (*M* = 23.5; *SD* = 2.8); 20 were male and 40 female. Data was collected in three waves: April 2015 (Wave 1), May and June 2015 (Wave 2), and July and August 2015 (Wave 3). Participants were recruited via leaflets and posters distributed in different buildings of the University of Freiburg. Potential participants were invited to write us an e-mail so that we could call them back and screen them for eligibility. Individuals were included if they currently exercised at least once a week for at least 30 min. In addition, since we intended to investigate activities that are self-directed and therefore might be affected by momentary states, participants had to indicate that they did not exercise solely because they felt obliged to do so because of other people (athletic clubs, parents, friends). Individuals who were competitive athletes or were enrolled in a sports science degree program or had undergone psychiatric therapy/treatment in the previous 12 months were excluded from the study. Individuals who met the eligibility criteria were scheduled for the initial laboratory meeting at the university. Participants received a financial compensation of 20 € after the end of the study.

### Procedure

The study protocol was reviewed and approved by the institutional ethics committee. For data collection, we used an electronic diary via smartphones (“Google Nexus 5”: LG, Seoul, South Korea), using the app “movisensXS” (movisens GmbH, Karlsruhe, Germany). We chose to collect data over a long period (20 working days) in order to obtain generalizable data. In addition, we assumed that methododological reactivity, the possibility that participants change their usual beahvior because it is being monitored, would be less likely if the data collection period was longer. At the initial laboratory meeting, participants gave their written consent, completed a questionnaire assessing demographic characteristics, received the necessary equipment, and were instructed on how to operate the smartphones. They were also informed about the following facts and procedures during the study period of 20 working days (Monday to Friday): the calling and Internet capabilities of the smartphones were disabled. The participants were asked to keep the phones switched on throughout the period. An acoustic signal prompted the participants to make an entry of data collection in the electronic diary at four random measurement points per day within four pre-programmed time windows: in the morning (between 9:30 and 10:30 a.m.), around midday (between 12:30 and 1:30 p.m.), in the evening (between 4:30 and 5:30 p.m.), and at bedtime (between 10:00 and 11:00 p.m.). If the participant made no entry, the smartphone beeped again after a few minutes. The participants could also decline to make entries or delay them by up to 20 min. During class or in the library, the students could set the phones to a vibration mode. The app was programmed to display the questions and provide the response choices on the smartphone screen. The completion of the electronic diary entry took about 2–3 min. Affect scores were assessed at all measurement points, four times a day for 20 working days. At bedtime, participants additionally provided data on the amount of time they had spent exercising that day. After the data collection period, participants were invited to the laboratory again to return the smartphones and to receive the financial compensation. Data were stored on the phone and were downloaded to a server after the end of the study.

### Measures of Affect

There are many different definitions and specifications of mood, emotions, and affect. In the current paper, we follow Panteleimon Ekkekakis’s definition of affect as an “immediate, uncontrollable, automatic response” (p. 95), a “pleasant or unpleasant feeling that does not require any prior cognitive processing” (p. 95), with typical affects being the feeling of “pleasure vs. displeasure,” “energy vs. tiredness,” or “tension vs. calmness” (Hanin and Ekkekakis, 2014, p. 95). To assess the variability of affect in daily life, we used a short scale for assessing affect developed and validated for EMA by Wilhelm and Schoebi (2007) and based on the Multidimensional Mood Scale (MDMQ; Steyer et al., 1997). The six-item scale measures three dimensions of affect with two bipolar items per dimension: valence (well–unwell; content–discontent), energetic arousal (tired–awake; full of energy–without energy), and calmness (agitated–calm; relaxed–tense). Participants had to rate the items on a seven-point scale on the basis of the statement “At the moment, I feel …” They provided the answers by choosing a number from 0 to 6 on the display.

### Measures of Naturally Occurring Exercise

Naturally occurring exercise was measured as a continuous variable (exercise duration) by the item “How many minutes in total have you been engaging in exercise today?” Participants could type in the number of minutes. At the initial laboratory meeting, participants were explained the difference between instrumental “everyday life activities” (e.g., stair climbing, going to work by bicycle) and activities that are defined as “exercise behavior” (including all classical sports and health-related exercises; Fuchs et al., 2015). Most of the studies mentioned above used accelerometers to assess physical activity objectively. Since we were not interested in daily life physical activity but in naturally occurring planned exercise done for its own sake (sports) or for health-related reasons, which will probably occur only every 2–3 days, it was not necessary or suitable for our participants to wear accelerometers during the entire period of data collection (20 days). This would have placed another burden on the participants, possibly reducing compliance. Therefore, we relied on EMA self-reports to assess naturally occurring exercise duration.

### Data Analysis

Since we wished to observe exercise duration as it occurs in real life, the time of the day for exercising will differ from individual to individual and from day to day. In addition, it is hard to assess affect directly before and after episodes of exercise in real life since the event “exercise duration” occurs too infrequently in real life (e.g., three times a week). We therefore decided to assess affect four times a day at fixed time points and used the mean level of all four affect measurements from the whole day to clarify whether there was an association between affect and exercise duration on a day level. Plus, we used the morning affect measurement point to predict exercise duration on that day (Carels et al., 2007) and the evening affect measurement point to predict affective reactions from exercise duration that day.

The data from our study had a nested structure, with 20 days nested within 60 persons. Therefore, we estimated multilevel models using HLM 7.0 (Raudenbush et al., 2011). Multilevel models can differentiate between within-subject effects (level 1: day level) and between-subject effects (level 2: person level). We used restricted maximum likelihood estimations for the analysis and set the α-level of the tests to *p* < 0.05. Prior to analysis, within-person predictors were person-mean centered (Snijders and Bosker, 2011). We started by ckecking if the affect assessment data of the different days were not correlated. If participants reported similar affect scores on two consecutive measurement occasions, there is a risk of underestimation of the within-subject variability (Schwartz and Stone, 1998). Correlations are provided in Supplementary Material.

Model 1: Association between exercise duration and affective states on a day level

In the first analysis, we started by estimating the intra-class coefficient (ICC) in a model in which exercise duration is not modeled as a function of another variable. Second, we entered the predictor variables valence, energetic arousal, and calmness (day mean) into the model on level 1. We also entered gender into the model on level 2 as a control variable.

Level 1 Model:

$${Exercise duration}_{\mathrm{ti}}\;=\;b_{0i}+b_{1i}{}^{*}{\left(\mathrm{Valence}\right)}_{\mathrm{ti}}+b_{2i}{}^{*}{\left(Energetic arousal\right)}_{\mathrm{ti}}+b_{3i}{}^{*}{\left(\mathrm{Calmness}\right)}_{\mathrm{ti}}+r_{\mathrm{ti}}$$

Level 2 Model:

$$\begin{array}{c} b_{0i}=\gamma_{00}+\gamma_{0i}{}^{*}(\mathrm{Gender})+\mu_{0i}\\ b_{1i}=\gamma_{0}+\gamma_{11}+\mu_{1i}\;\\ b_{2i}=\gamma_{20}+\gamma_{21}+\mu_{2i}\;\\ b_{3i}=\gamma_{30}+\gamma_{31}+\mu_{3i}\; \end{array}$$

The level 1 model represents the participant’s responses (subscript i) to the exercise duration item on the day level (subscript t). Exercise duration is a function of the intercept *b*_0i_ and the three level 1 predictors (*b*_1i_, *b*_2i_, *b*_3i_). *b_1_*_i_ is the effect of the within-person valence, meaning the increase in exercise duration with every increase of valence. *b*_2i_ is the within-person effect of energetic arousal, and *b*_3i_ is the within-person effect of calmness. The error for level 1 is given by *r*_ti_. Level 2 expresses the between-subject effect, including the fixed effects γ_00_ of the averaged intercepts and slopes across all participants and the covariate γ_0i_, which displays the effect of gender on exercise duration. This level also includes the random effect μ_0i_.

Model 2: Subsequent exercise duration as predicted by affective states

In the second model, we entered the affect variables into the model like in model 1, but instead of using the day mean for each affect dimension, we used only the affect data collected at the morning assessment point.

Model 3: Subsequent affective states as predicted by exercise duration

In the third model, we started again by estimating the ICC but this time in three different models without predictors. Valence, energetic arousal, or calmness functioned as the respective outcome variables in the three models. In a second step we entered exercise duration as a level 1 predictor, and in a third step we entered gender on level 2. On the basis of these variables, we estimated whether valence, energetic arousal, and calmness (intercept) varied significantly as a function of gender.

Level 1 Model:

$$\mathrm{Affect}{\left(Valence/Energetic arousal/Calmness\right)}_{\mathrm{ti}}=b_{0i}+b_{1i}{}^{*}{\left(\mathrm{Exercise}\mathrm{duration}\right)}_{\mathrm{ti}}+r_{\mathrm{ti}}$$

Level 2 Model:

$$\begin{array}{c} b_{0i}\;=\;\gamma_{00}+\gamma_{01}{}^{*}(\mathrm{Gender})+\mu_{0i}\\ b_{1i}\;=\;\gamma_{10}+\gamma_{11}+\mu_{1i}\; \end{array}$$

The Level 1 model calculated the within-subject effects. It represents the participant’s responses (subscript i) on one of the affect subscales on a day level (subscript t). The affect subscale is a function of the individual intercepts *b*_0i_ and *b*_1i_, which is the effect of the within-person exercise duration, meaning the increase in affect with every increase of exercise duration. On Level 2, γ_01_ displays the effect of gender on affect.

## Results

### Descriptive Analysis

The sample with *N* = 60 participants provided 1072 data points for model 1 (association), 1159 data points for model 2 (exercise duration as predicted by morning affect), and 1082 data points for model 3 (evening affect as predicted by exercise duration). The overall average exercise duration across all subjects per day was *M* = 38.7 min (*SD* = 50.9 min), with a range from 0 to 300 min. Overall, the participants exercised on 48.2% of the days (exercise duration > 0 min), with a range from 2 to 17 days (out of 20 days) per person. The average daily levels for valence, energetic arousal, and calmness were 9.8 (*SD* = 1.7), 8.6 (*SD* = 1.7), and 9.3 (*SD* = 1.5), respectively, representing a medium levels of affective states.

### Test of Hypotheses

Model 1: Association between naturally occurring exercise duration and affective states on a day level

To test whether affect is associated with exercise duration on a day level, we first estimated the ICC of exercise duration (*ρ_I_* = 0.16). It indicated that 84% of the variability in exercise duration was attributed to within-person differences and only 16% to between-person differences. This demonstrates that exercise duration shows substantial within-person variation on a day level, which requires a multilevel approach. Next, we entered the predictors into the model. The model is presented in **Table 1**. The random effects of the energetic arousal and calmness slope were not significant, so they were removed. The slope of valence varied between persons (*p* < 0.001). Exercise duration was almost significantly associated with gender (*p* = 0.050), indicating that men exercised more than women. We also found that exercise duration was significantly positively associated with valence (*p* = 0.003) but not with energetic arousal (*p* = 0.239), indicating that good feelings were related to a longer exercise duration. The association between exercise duration and calmness was negative but not significant (*p* = 0.141).

**Table 1:** Predicting exercise duration by valence, energetic arousal, and calmness on a day level.

Fixed effects					Random effects			
	Est	*SE* (df)	*t*-ratio	*p*-value	Variance estimate	*SD*	*X*(df)	*p*-value
Intercept	22.88	8.38 (58)	2.73	0.008	443.02	21.05	240.58 (57)	<0.001
Gender	11.77	5.89 (58)	1.99	0.050				
Valence	2.02	0.65 (59)	3.11	0.003	5.23	2.29	105.12 (58)	<0.001
Energetic arousal	0.44	0.37 (666)	1.18	0.239				
Calmness	-0.63	0.43 (666)	-1.48	0.141				

Model 2: Subsequent exercise duration as predicted by affective states

In model 2, the three dimensions of affect that were assessed in the morning functioned as predictors of exercise duration that day. The random effects of the three slopes were not significant, so they were removed. The model is presented in **Table 2**. We found that exercise duration was again almost significantly associated with gender (*p* = 0.052) and with energetic arousal (*p* = 0.045) but not with the other affect dimension (valence: *p* = 0.736; calmness: *p* = 0.740).

**Table 2:** Prediction of daily exercise duration by valence, energetic arousal, and calmness in the morning.

	Fixed effects				Random effects			
	Est	*SE* (df)	*t*-ratio	*p*-value	Variance estimate	*SD*	*X*(df)	*p*-value
Intercept	22.59	8.86 (58)	2.55	0.013	406.96	20.17	243.12 (58)	<0.001
Gender	12.15	6.13 (58)	1.98	0.052				
Valence	0.46	1.37 (916)	0.34	0.736				
Energetic arousal	1.55	0.77 (916)	2.01	0.045				
Calmness	-0.34	1.03 (916)	-0.33	0.740				

Model 3: Subsequent affective states as predicted by exercise duration

The last model estimated the extent to which exercise duration predicts affect in the evening. The results for the ICC were *ρ_I_* = 0.22 for valence, *ρ_I_* = 0.23 for energetic arousal, and *ρ_I_* = 0.24 for calmness, indicating that 78, 77, and 76% of the affect subscale’s variance, respectively, was caused by within-person variation. The model is presented in **Table 3**. In the valence model, the random effects of exercise duration varied between persons (*p* = 0.043), whereas the random error terms of exercise duration in the other models had to be removed. Valence in the evening was significantly predicted by gender (*p* < 0.001) and by exercise duration (*p* = 0.009), indicating that men felt better and more content in the evening than women and that participants felt better and more content the longer they had exercised that day. Energetic arousal and calmness were both significantly predicted by gender (*p* = 0.002; *p* = 0.003) but not by exercise duration (*p* = 0.954; *p* = 0.144). The random error terms of the slopes in these models were not significant, so they were fixed. Gender differences indicated that men had significantly higher scores on all three affect subscales.

**Table 3:** Prediction of valence, energetic arousal, and calmness by exercise duration.

	Fixed effects				Random effect			
	Est	*SE* (df)	*t*-ratio	*p*-value	Variance estimate	*SD*	*X*(df)	*p*-value
**Model: valence**								
Intercept	8.49	0.41 (58)	20.48	<0.001	0.94	0.97	285.94 (58)	<0.001
Gender	1.13	0.28 (58)	4.08	<0.001				
Exercise duration	0.00	0.00 (59)	2.69	0.009	0.01	0.00	78.78 (59)	0.040
**Model: energetic arousal**					
Intercept	5.59	0.52 (58)	10.73	<0.001	1.52	1.23	314.93 (58)	<0.001
Gender	1.17	0.35 (58)	3.302	0.002				
Exercise duration	0.00	0.00 (981)	0.06	0.954				
**Model: calmness**					
Intercept	8.34	0.49 (58)	17.03	<0.001	1.36	1.17	342.82 (58)	<0.001
Gender	1.078	0.34 (58)	3.15	0.003				
Exercise duration	0.01	0.00 (981)	1.46	0.144				

## Discussion

This ambulatory assessment study analyzed the bi-directional effects of naturally occurring exercise duration on affective states and vice versa. The within-subject associations revealed three important findings: first, only positive affective valence was positively associated with exercise duration on a day level; second, only energetic arousal in the morning predicted subsequent exercise duration that day; and third, exercise duration only predicted higher positive affective valence in the evening. Thus, our hypotheses were only partly confirmed since we could only find effects on one of the three dimensions (positive affective valence, calmness, energetic arousal) for each analysis. The significant effects of gender in the analyses might be due to the fact that we had an unequal distribution of gender in our sample. The findings support the hypothesis that positive affective valence is positively related to naturally occurring exercise. This is in line with other findings (Gauvin et al., 1996; Carels et al., 2007; Dunton et al., 2009, 2014; Kanning and Schlicht, 2010; Schwerdtfeger et al., 2010; von Haaren et al., 2013). However, our data did not support the hypothesis that the feeling of being energized (energetic arousal) or relaxed (calmness) is related to exercise duration. However, this result is consistent with findings from Dunton et al. (2009), who also did not find a significant positive association between energetic arousal and physical activity among middle-aged and older adults.

To obtain evidence on the temporal sequence and direction of the association between naturally occurring exercise and affect, we investigated whether affect predicted exercise duration in the morning and whether exercise duration predicted affect in the evening. Findings revealed that only energetic arousal in the morning predicted exercise duration. Participants exercised more on days on which they felt energized and awake in the morning. This finding is in line with results from Dunton et al. (2014), who found that an energetic feeling predicts physical activity in children. Contrary to findings from other ambulatory assessment studies (e.g., Schwerdtfeger et al., 2010; Dunton et al., 2014), we did not find that positive affective valence predicts exercise. These studies revealed that positive feelings predict subsequent physical activity. However, Schwerdtfeger et al. (2010) assessed “positive activated affect,” measured with six items: lively, awake, active, powerful, dynamic, and happy. It comprises positive affective valence as well as energetic arousal. The results indicate that it might be wise to assess the two dimensions separately.

With regard to our third hypothesis, the data confirmed previously published findings by showing that the more participants exercise, the better and more content they feel (positive affective valence) in the evening of that respective day. This is in line with results from Gauvin et al. (1996), Carels et al. (2007), Mata et al. (2012), von Haaren et al. (2013), and Kanning et al. (2015), who found a positive association between physical activity and subsequent positive affect. In contrast to our findings, some of these studies also reported significant associations between physical activity and energetic arousal as well as physical activity and calmness. However, most former studies analyzed the association between momentary affect and momentary activity in daily life. They did not assess whether physical activity or exercise predict affective states in the evening. It may be that the association between affect and activity depends on the assessment interval. It could be that feelings of relaxation and power will disappear as time goes by. Further studies are needed to assess the predictive power of physical activity and exercise on subsequent affect in different time frames.

Our main results are that participants exercised more on days on which positive affective valence was high and that they showed higher positive affective valence in the evening the more they had exercised that day. Plus, the more energized and awake participants felt in the morning, the more they exercised that day. Our explanation for the positive association between positive affective valence and exercise is that positive affective valence facilitates exercise behavior by motivating the individual. According to Baumeister et al. (2007), affect acts as a short-term, intra-individual predictor of behavior since automatic positive affective valence leads to an increase in goal pursuit. It may be assumed that when individuals experience positive affect, they become motivated to reach their long-term health goals, thus facilitating exercise initiation. Positive affect has also been shown to counteract ego-depletion (Ren et al., 2010), which is a state of a diminished self-control resource (Baumeister, 2002). Ego-depletion follows after a person has carried out tasks that require self-control. In daily life, it may be the case that self-control is low due to daily self-control demands that an individual is confronted with, and as a consequence the individual may no longer be capable of initiating a behavior that requires self-control. Exercise behavior depends on self-control since it is related to long-term health goals. Positive affect might compensate for diminished self-control. Feeling energized in the morning might have the same effect. It may be an indicator of high self-control on that day. In sum, affect may play a role in the acute initiation and duration of exercise, because the current state of the individual is a predictor of current behavior. However, since data analyses have shown that the more participants exercise, the higher their positive affective valence is in the evening, it also seems plausible to assume that the relationship works the other way around.

### Strength and Limitations

We conducted an EMA study to investigate within-subject associations between momentary affective states and naturally occurring exercise that provides high methodological standards (Kanning et al., 2013). An ambulatory assessment allows for an investigation with high ecological validity (Shiffman et al., 2008; Trull and Ebner-Priemer, 2013). Since we collected data for 20 days, we assume that the data accurately reflected the daily lives of the individuals. The assessment of both affect and naturally occurring exercise in real life led to a high external validity of our data. Another strength of our study is that we used a multidimensional measure of affect and thus took not just valence but also arousal and calmness into account. Wilhelm and Schoebi (2007) have shown that this three-dimensional assessment of affect is suitable and adequate for registering fluctuations in affect in real time (cf. Schimmack and Grob, 2000).

Despite these benefits, the decision to employ EMA comes at the expense of a limited experimental control of confounding variables. External factors (e.g., doctor’s appointments) beyond affective states might have an impact on exercise duration. In addition, other events besides exercise (e.g., social conversations) might have an impact on affect in the evening. A methodological limitation of our study was that normality of the dependent variable cannot be assumed, since natural occurring exercise duration is a skewed variable. Yet another limitation of our study was that our sample consisted of university students. Thus, generalizations based on these results need to be made with caution, because they may be affected by selection bias. Furthermore, it may be that only individuals who were especially motivated to exercise regularly were attracted by our posters and leaflets, which is supported by the high values of naturally occurring exercises in the sample.

### Implications for Future Research

Future research should investigate the bi-directional effects of affect on naturally occurring exercise and vice versa in a more controlled way. Most of all, it is important to create a design in which affect is assessed immediately before and after a person exercises in daily life (e.g., with an activity-triggered design). This allows identification of the temporal sequence of the association and more precise assessment of the underlying dynamic relationship between affective states and naturally occurring exercise. In addition, due to dense assessments there are fewer influences that might have an impact on affect or exercise as the dependent variable. It would also be interesting to examine the effect of naturally occurring exercise on affect and vice versa with varying time intervals (e.g., after 1 h, after 3 h) to examine how long the effects of affect or exercising last. Plus, exercise days could be compared to non-exercise days regarding the stability or enhancement of positive affective valence over the day. Experimental approaches will be needed to enable causal inferences. As a means of obtaining a randomized controlled trial, it might be useful to randomly assign individuals to activity conditions (Liao et al., 2015). To validate the self-report data of exercise duration, future studies should combine self-reports and objective data (accelosensors). Since affect is not the only variable to influence exercise behavior in daily life, the interplay between affective states and other important variables, such as intentions, should be taken into account in future studies. In addition, future research should investigate the bidirectional effects of affective states and exercise in initiating exercise bahavior in individuals who do not yet exercise. Our study emphasizes the importance of analyzing within-subject effects when investigating the relationship between naturally occurring exercise and affective states. Future intervention studies that aim to increase naturally occurring exercise should take situational variables such as momentary affective states into account. Furthermore, strategies for increasing positive affective valence should be considered as means of enhancing naturally occurring exercise.

## Conclusion

The health message of this study clearly states that naturally occurring exercise behavior is associated with positive affective valence on a day level and with positive affective valence in the evening. The findings demonstrate that it would be worthwhile to focus more on within-subject analyses when analyzing the dynamic relation between affective states and naturally occurring exercise. Whether the affective state precedes the exercise behavior or is rather a response to exercise behavior is not quite clear on the day level. However, either way it might affect exercising in daily life, because positive affective states preceding exercising might facilitate the initiation and duration of exercising, whereas affective states following exercising might affect future exercise behavior by having an impact on expectations. We propose that inducing positive affect before exercising and, for example, a conscious focusing on positive affect after exercising could increase naturally occurring exercise. In addition, “feeling energized and awake” might be an important precondition for exercising. Momentary affective states should be taken into account in the creation of effective interventions to foster habitual physical exercise.

## Author Contributions

AS contributed to the conception and the design of the study. She analyzed and interpreted the data and did a first draw of the paper. She did a final approval. MK analyzed and interpreted the data and was revising the paper critically for important intellectual content. She did a final approval. RF contributed to the conception and design of the study. He was revising the paper critically for important intellectual content and did a final approval.

## Conflict of Interest Statement

The authors declare that the research was conducted in the absence of any commercial or financial relationships that could be construed as a potential conflict of interest.
