# Supplementary material for: The Bidirectional Effect between Momentary Affective States and Exercise Duration on a Day Level
Source: Front Psychol. 2016 Sep 21;7:1414. doi: 10.3389/fpsyg.2016.01414 (PMC5030271; doi:10.3389/fpsyg.2016.01414)
Supplement: Supplementary file 1 [file Table_1.DOCX]

Supplementary Material

**The bidirectional effect between momentary affective states and exercise duration on a day level**

**Anna Schöndube1*, Martina Kanning2, Reinhard Fuchs1**

*** Correspondence:** Anna Schöndube, Institute of Sport and Sport Science, Department of Sportpsychology, Freiburg University, Schwarzwaldstraße 175, Freiburg, 79117, Germany.

**Table S1. Correlations between affect measurement occasions of two consecutive days.**

| Correlations between days | **Valence** | **Energetic Arousal** | **Calmness** |
| --- | --- | --- | --- |
| 1/2 | .173 | .069 | -.003 |
| 2/3 | .078 | -.096 | .053 |
| 3/4 | -.193 | -.304^*^ | -.034 |
| 4/5 | -.146 | -.158 | .057 |
| 5/6 | .232 | .158 | .032 |
| 6/7 | .087 | .407^**^ | -.073 |
| 7/8 | 210 | .  332^*^ | .135 |
| 8/9 | .079 | -.007 | .303^*^ |
| 9/10 | .194 | .196 | .094 |
| 10/11 | .110 | -.087 | .223 |
| 11/12 | .280 | .270 | .385^*^ |
| 12/13 | .267 | -.040 | .453^**^ |
| 13/14 | -.121 | .106 | -.280 |
| 14/15 | .311 | .136 | .012 |
| 15/16 | .021 | -.111 | -.219 |
| 16/17 | -.020 | .255 | .202 |
| 17/18 | .167 | .121 | .279 |
| 18/19 | .255 | .434^*^ | .038 |
| 19/20 | -.065 | -.153 | .213 |

** p* < .05; ***p* < .01
